# Compare first, evaluate later: Upending the neuroscience of choice

**DOI:** 10.1371/journal.pbio.3003295

**Published:** 2025-08-08

**Authors:** Benjamin Y. Hayden

**Affiliations:** 1 Neurosurgery, Baylor College of Medicine, Houston, Texas, United States of America; 2 Electrical and Computer Engineering and Linguistics, Rice University, Houston, Texas, United States of America

## Abstract

In standard models of economic choice, we evaluate each option separately and then compare their values. This Primer discusses a new study in PLOS Biology showing that, in orbitofrontal cortex, we compare before evaluating, challenging theories about how we choose.

## Teaser

In standard models of economic choice, we evaluate each option separately and then compare their values. A new study in PLOS Biology shows that, in orbitofrontal cortex, we compare before evaluating, challenging theories about how we choose.

Would you rather have a chocolate cupcake or a healthy pear for dessert? A luxury vacation with your family or a sensible used car of the same price? An afternoon nap or an afternoon reading an exciting new paper by a favorite neuroscientist? Nearly all real-world economic choices consist of bundles of dissimilar features or attributes. The art in making a choice, then, comes in figuring out how to compare multi-attribute goods. A new study in PLOS Biology by Perkins and Rich suggests our standard theories about how this happens may have a key feature backwards [1].

Since the birth of the field, 25 years ago, a central dogma in neuroeconomics has been that we combine all attributes into a single value first, then compare the values of the options and select the best [2]. The evaluation-first principle makes a lot more sense than the alternative, a comparison-first paradigm. Evaluation-first leverages the key benefit of an abstract value system, the fact that it allows for direct comparison of any incommensurate goods. Plus, it is a lot simpler to implement. If we compare across dimensions first, we are faced with the need to somehow integrate the results of qualitatively different comparisons, occurring in different dimensions, with separate scales.

For most of the past 25 years, the neural evidence was seen to argue strongly in favor of the evaluation-first paradigm. Indeed, the central research goal in neuroecomics has been the search for these post-evaluation, pre-comparison abstract value signals [2]. A series of landmark studies identified the orbitofrontal cortex (OFC), which has become central to most theories of multi-attribute choice as the center of cross-dimensional integration [3]. Critically, neurons in the OFC have firing rates that correlate with the relative values of options regardless of which features of those options drive the value. As a result, the OFC is generally seen as a kind of central furnace of choice, where all features are melted together into a single composite value signal, creating a feature-independent alloy that is the ideal input for comparison. Indeed, the idea that the OFC computes abstract values and only then compares them, has become something of a central dogma for neuroeconomics [3].

But the past several years have seen a series of challenges to that dogma [4]. Among other things, the idea of a special role for the OFC has been challenged by evidence for value signals in other regions, including studies showing value in nearly every part of the brain [5,6]. If value representations can be found in nearly every part of the brain, that raises troubling questions for the dogma, including core questions like whether the idea of a specialized value center is well-founded.

At the same time, psychologists have been collecting more and more evidence that many—perhaps most—of our decisions are driven by heuristics, most of which do not involve a specific value calculation step [7]. Indeed, heuristics are particularly likely to be used in the case of multi-attribute choice. For example, a classic use case in heuristics is the choice between apartments differing in square footage, location, amenities, and so on. How can we make choices without evaluate-then-choose? It turns out there are many simple ways. For example, in the lexicographic heuristic, we identify the most important attribute then choose the option with the best value on that attribute. In elimination by aspects, we set a threshold for one attribute, then eliminate subthreshold options, then move to successively less important attributes. Critically, heuristics can produce choices so accurate that they are more or less indistinguishable from optimal ones except in rare edge cases. We use heuristics because they are cognitively cheaper—they do not require nearly as much mental effort, and, while they do not provide an optimal solution, they provide choices that are close enough. In other words, while evaluate-first is simple and elegant for us, as scientists, to model, it is much more onerous than heuristic alternatives.

In a new study by Perkins and Rich [1], monkeys made a series of multi-attribute choices in which options differed on the dimensions of sweetness and probability. Neurons in the OFC were found to code one of the two attributes, rather than integrated values. Those value codes are relative, meaning that they encode the difference in values for those two dimensions for the two offers, and depend on which target the subject is looking at. This is crucial, because a relative valuation is mathematically equivalent to a value comparison, and, with a simple rectification step, is equivalent to choice. In other words, these OFC neurons are doing comparison before evaluation. That violates the dogma that evaluation must precede comparison. This study, therefore, builds on a small but growing set of findings that cannot readily be accounted for within the evaluate-first paradigm [8,9].

**Fig 1:**
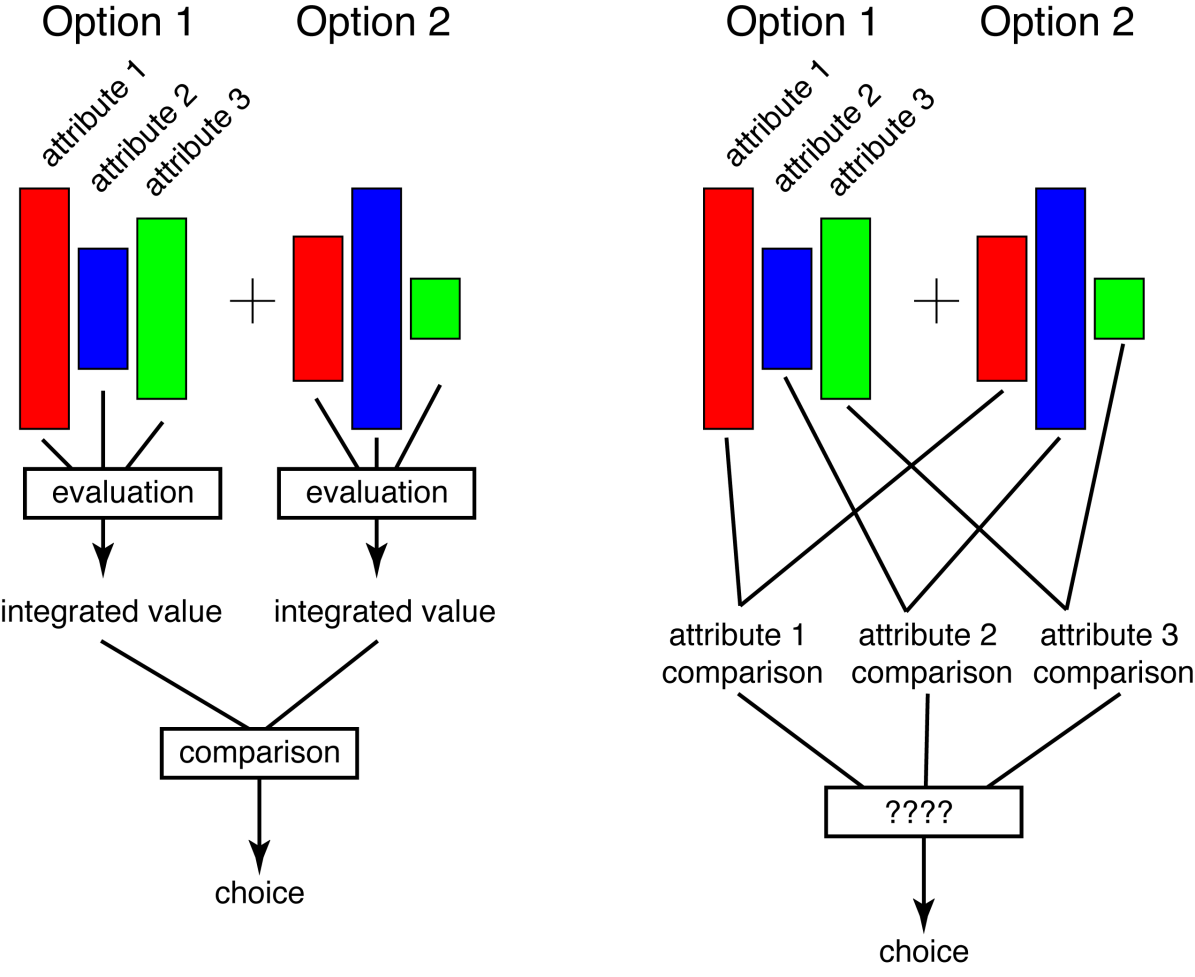
In evaluation-first choice, the decision-maker combines information across attributes within each option (evaluation) and then takes the results of that evaluation step (abstract values) and compares them (comparison). In comparison-first choice, the decision maker compares each attribute separately for each object and then performs an as-yet-unidentified step to determine which option to choose. Comparison-first choice may be preferred because it avoids a costly or difficult evaluation step.

Why would the brain not do evaluate first if it is the more efficient way? Speculatively, maybe the bottleneck in economic choice is the coordination of information. Maybe getting two specific pieces of information to combine in a particular way at a particular time and routed to a specific neuron might be extremely difficult, like finding a needle in a haystack. So maybe the brain instead has developed ways of making choice that are pretty good—not perfect—that avoid the need for perfect informational routing. Maybe computing a single fully integrated value signal is a lot harder than it might seem, and the brain has evolved a way to avoid it. And that may explain why heuristics are so ubiquitous. And that in turn may explain our often pretty good, but sometimes unfortunate biases, including poor choices, poor self-control, and associated mental health and lifestyle problems [10] (Fig 1).

## Abbreviation:

OFC: orbitofrontal cortex
